# Those Who Teach, Do

**DOI:** 10.1007/s13187-025-02640-z

**Published:** 2025-05-07

**Authors:** Beatrice T. B. Preti

**Affiliations:** 1 Department of Oncology, Western University, London, ON Canada; 2 Department of Haematology & Medical Oncology, Emory University, Atlanta, GA USA; 3 School of Health Professions Education, Maastricht University, Maastricht, Netherlands

**Keywords:** Medical education, Oncology education, Teaching

“Those who can, do. Those who can’t, teach.”

These words echo in my mind often. I first heard Shaw quoted in jest by a mentor who watched me struggle to memorise oncology trials, while picking up teaching techniques and research strategies with ease. Yet the sentiment is repeated more often by those around, and not always with kindness. As a medical educator, it is easy to lose sign of the path, the purpose, of our work amid bureaucracy, institutional politics, and medical culture. As a medical oncologist, it is even harder, homed in a world comprised of statistics, pharmacology, and dollar signs, yet persistently shrouded in death.

All too often, I wonder what it is all for.

At a recent medical education conference, I had the honour of witnessing two of my junior medical students present a project I had coached them through. The idea was their own, and they had meticulously worked through several PDSA cycles, culminating in a conference abstract, poster, and even a manuscript. I was delighted to see the interest their project generated, including questions and praise from several high-profile international educators.

During the session, one of my students texted me, “Wow this is very cool.” She was also thrilled by the response to their work, and I realised I was witnessing, for the first time in my career, a medical learner’s academic awakening. All too often, we can get distracted or weighed down by the pressures of the work we do.

But do you remember that first budding of interest in medicine? Academia? The education process? What about the process of creating something new, a brain-child, and presenting it to the world? Remember the raw, unbridled joy that comes from discovery, sharing, learning, and growing. And then imagine seeing it in another for the first time.

One of my favourite classes I have ever taught is first-year clinical skills to medical students. There is something magical about the first time a future doctor palpates a pulse or auscultates breath sounds. It was the same unbridled joy my student was experiencing now, and I found myself grinning along with her, delighted by her delight.

In medical oncology, it is easy to give into despair, to lament the ever-looming threat of death and disability that haunts dark corners (and dark thoughts) of the cancer journey. In medical education, likewise, it is easy to get lost in the weeds, to wonder why we fight politics and bureaucracy, and why we do what we do.

It is for moments like this. And the realisation that we have the privilege to be part of something bigger than ourselves.

And, maybe, while those who can, “do,” maybe those who teach, also “do” something of their own.

